# Supplementary material for: Single molecule kinetics of bacteriorhodopsin by HS-AFM
Source: Nat Commun. 2021 Dec 10;12:7225. doi: 10.1038/s41467-021-27580-2 (PMC8664958; doi:10.1038/s41467-021-27580-2)
Supplement: Supplementary file 1 — Supplementary Information [file 41467_2021_27580_MOESM1_ESM.pdf]

Single molecule kinetics of bacteriorhodopsin by HS-AFM

Alma P. Perrino[1], Atsushi Miyagi[1] and Simon Scheuring[1,2,*]

[1] Department of Anesthesiology, Weill Cornell Medicine, New York, NY, USA

[2] Department of Physiology and Biophysics, Weill Cornell Medicine, New York, NY, USA

* correspondence to: sis2019@med.cornell.edu

Supplementary Information

Supplementary Figure 1

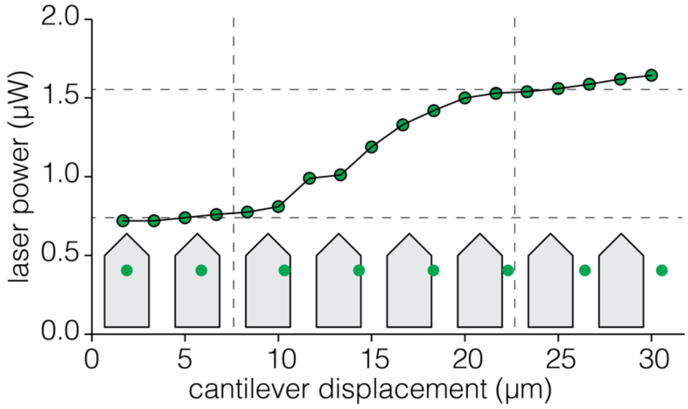

Supplementary Figure 1) Activating laser beam diameter determination. Using a 50-µm wide cantilever moving out of the laser beam while monitoring the laser power.

Supplementary Figure 2

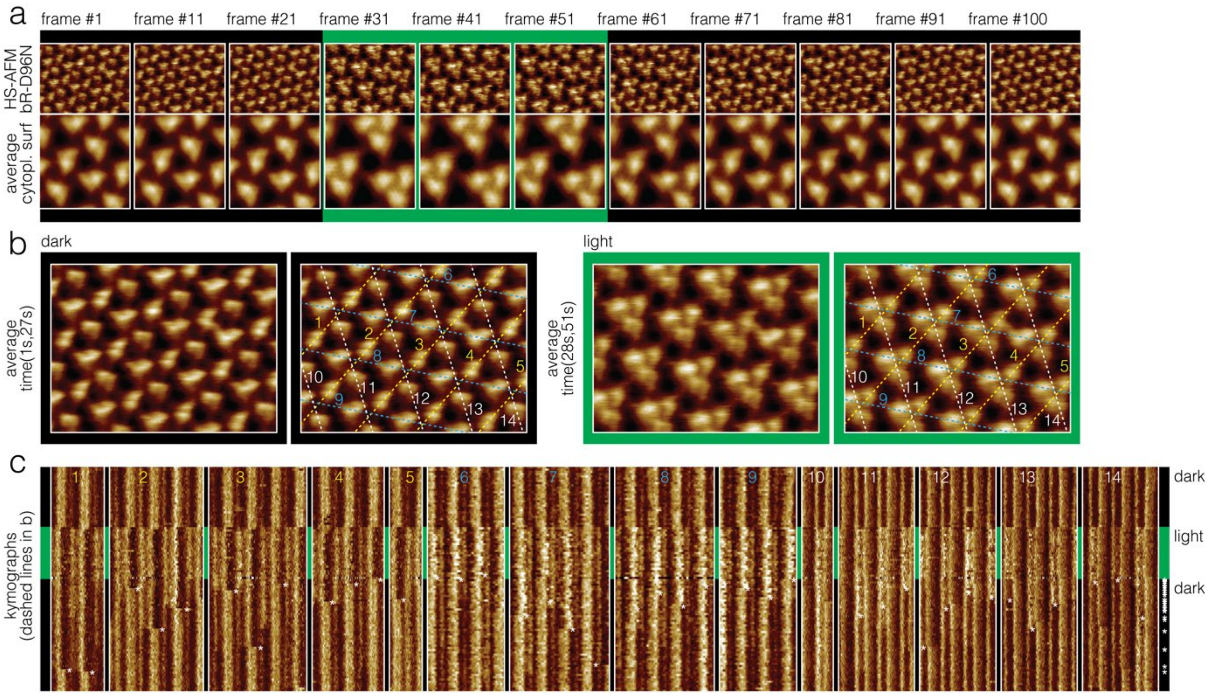

Supplementary Figure 2) Kymograph visualization of individual bR-D96N protomer dynamics. (a) High-resolution HS-AFM images (top) and corresponding correlation averages (bottom), as in main text figure 1e. Black and green backgrounds indicate dark and light conditions, respectively. **(b)** Average images of frames 1 to 27 (left) and frames 28 to 51 (right) with dashed lines indicating the positions of profiles in the below kymographs. **(c)** Kymographs along the dashed lines in (b). Light protocol is depicted at each side. White stars indicate the position at which the protomers return to the closed state.

Supplementary Figure 3

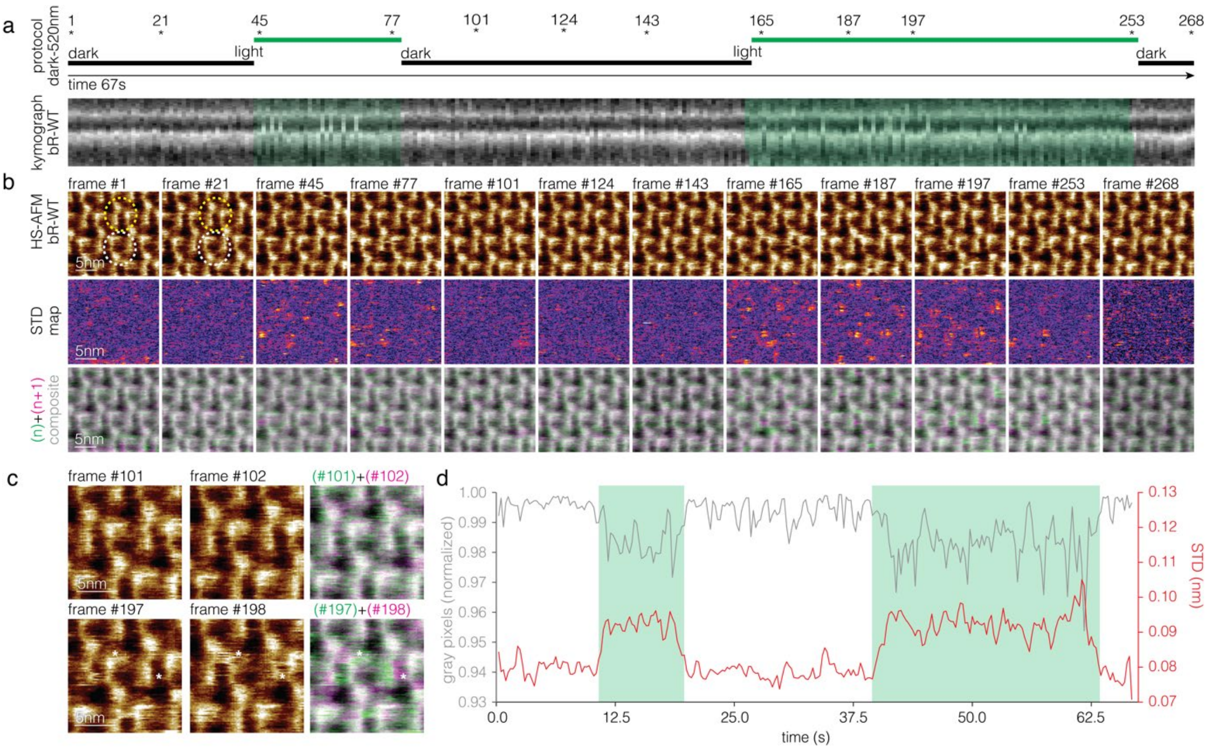

Supplementary Figure 3) bR-WT dynamics as reported in HS-AFM movies. (a) Light protocol (top panel). Kymograph extracted from the central scan line of a movie recorded at 4 frames per second (Supplementary Movie 4) (bottom). (**b**) HS-AFM images of bR-WT from Supplementary Movie 4. Maps of the standard deviation between consecutive frames as $SD = \sqrt{\frac{\sum(x_n - \bar{x})}{n-1}}$ (top). Composite of two consecutive frames (t, green) and (t+1, magenta) (middle). The overlay of green and magenta gives grey, where the two channels are identical or no conformational difference is found between the pixels in the consecutive frames. (**c**) Subsequent HS-AFM frames in dark conditions (#101-102) (top) and with light on (#197-198) (bottom). (**d**) Graph of the gray pixels extracted from the composite green-magenta movie of two consecutive frames. Grey pixels account for pixels with the same intensity in RGB magenta [255 0 255] and RGB green [0 255 0]. The number of grey pixels (grey trace) is significantly lower during light periods as compared to dark periods. The pixel difference values (red trace, right Y-axis) shows increased pixel variability between subsequent frames during activating light periods as compared to dark periods.

Supplementary Figure 4

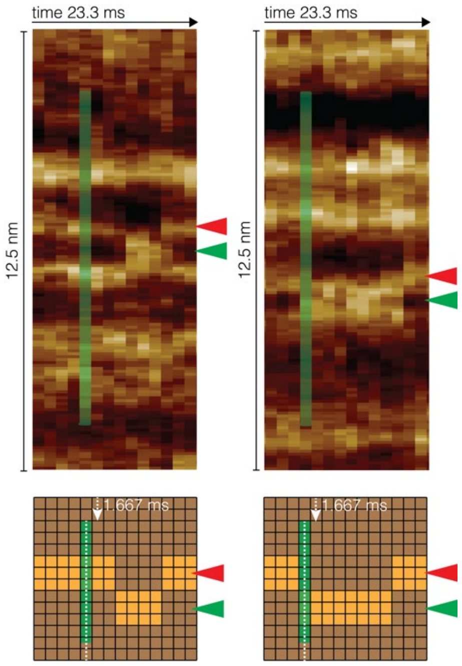

Supplementary Figure 4) Detail of the bR opening delay time analysis following short laser pulses. Top: Raw data display of 12.5 nm scan dimension and 23.3 ms kymograph of the conformational changes occurring in individual protomers. Green overlay indicates the signal from the photodiode. Bottom: Schematics of the pixels that constitute the kymograph image. Red and green arrowheads indicate the closed and open state positions, respectively. The duration of one scan line cycle is 1.667 ms.

Supplementary Figure 5

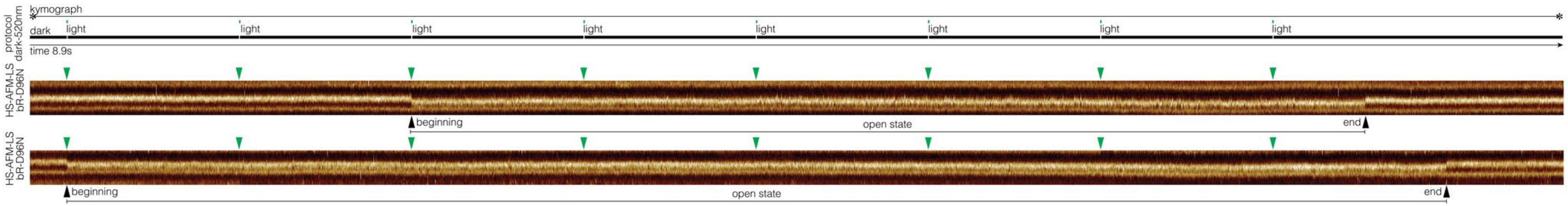

Supplementary Figure 5) HS-AFM-LS analysis of bR-D96N open state. Top: Light protocol, and bottom: kymographs of bR-D96N, in response to short activation light pulses (one 0.5 ms-pulse every second). The green arrowheads indicate the time of the activation light pulses.The black arrowheads indicate the beginning and the end of bR-D96N open states (as indicated).

Supplementary Figure 6

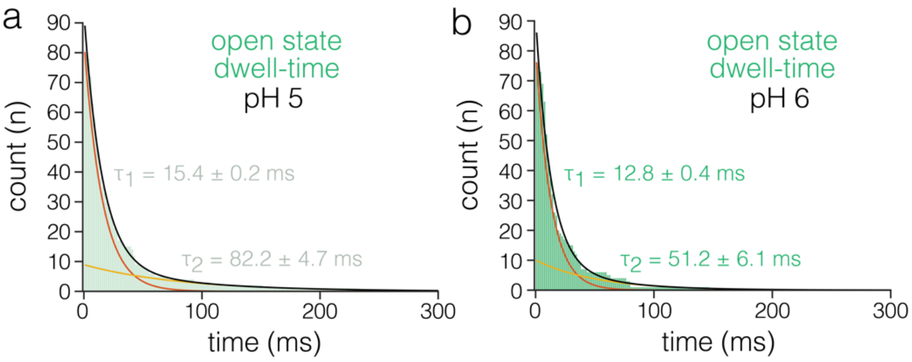

Supplementary Figure 6) Exponential fitting of open state dwell-time histograms of single molecule bR-WT at acidic pH. (a) At pH 5, and **(b)** at pH 6, the distributions were fitted using $y_{(x)} = C_1 e^{-x/\tau_1} + C_2 e^{-x/\tau_2}$. The fits result in, C_1=85±1, τ_1=15.4±0.2ms and C_2=9±1, τ_1=82.2±4.7ms at pH 5, and C_1=81±2, τ_1=12.8±0.4ms and C_2=10±2, τ_1=51.2±6.1ms at pH 6.

Supplementary Table 1

	**τ_1 (ms)**		**τ_2 (ms)**	
**pH 5**	15.4	± 0.2	82.2	± 4.7
**pH 6**	12.8	± 0.4	51.2	± 6.1
**pH 7**	14.2	± 0.1	-	-
**pH 8**	33.0	± 0.3	-	-
**pH 9**	36.0	± 0.2	-	-

Supplementary Table 1) Dwell times of the open state for bR-WT. In grey, the error of the fitting of the single exponential fit $y(x) = Ce^{-x/\tau}$ for pH>7 and double exponential fit $y(x) = C_1e^{-x/\tau_1} + C_2e^{-x/\tau_2}$ for pH 5 and 6 .

Supplementary Table 2

	**τ_1 (ms)**	
**pH 5**	2.5	± 0.1
**pH 6**	2.6	± 0.1
**pH 7**	2.9	± 0.1
**pH 8**	3.2	± 0.1
**pH 9**	2.4	± 0.1

Supplementary Table 2) Dwell times of the opening delay time for bR-WT. In grey, the error of the fitting of the single exponential fit $y(x) = Ce^{-x/\tau}$.
