# Supplementary material for: Single molecule kinetics of bacteriorhodopsin by HS-AFM
Source: Nat Commun. 2021 Dec 10;12:7225. doi: 10.1038/s41467-021-27580-2 (PMC8664958; doi:10.1038/s41467-021-27580-2)
Supplement: Supplementary file 3 — Description of Additional Supplementary Files [file 41467_2021_27580_MOESM3_ESM.docx]

Description of Additional Supplementary Files

File name: Supplementary Movie 1

Description: HS-AFM movie of two bacteriorhodopsin D96N patches exposing the cytoplasmic (left) and the extracellular (right) surfaces. The dark and green light activation periods are indicated by the alternating black and green bar below the movie. Insets: Correlation averages of the cytoplasmic and extracellular surfaces, respectively. Movie acquisition parameters: 100nm / 300pixels, 1 frame / second.

File name: Supplementary Movie 2

Description: HS-AFM movie of the cytoplasmic side of bacteriorhodopsin D96N exposed to a single light activation period indicated by the alternating black and green rectangle on the right bottom of the movie. Inset: Correlation average of the cytoplasmic surface. Movie acquisition parameters: 50nm / 300pixels, 1 frame / second.

File name: Supplementary Movie 3

Description: HS-AFM movie of the cytoplasmic side of bacteriorhodopsin D96N exposed to five light activation periods indicated by the alternating black and green bar below the movie. Inset: Correlation average of the cytoplasmic surface. Movie acquisition parameters: 70nm / 300pixels, 1 frame / second.

File name: Supplementary Movie 4

Description: Left: HS-AFM movie of the cytoplasmic side of bacteriorhodopsin WT exposed to two light activation periods indicated by the alternating black and green bar below the movie. Middle: Standard deviation map over two consecutive frames Right: Composite of two consecutive frames (t, green) and (t+1, magenta). The overlay of green and magenta gives grey, where the two channels are identical or no conformational difference is found between the pixels in the consecutive frames. Movie acquisition parameters: 50nm / 300pixels, 4 frames / second.

File name: Supplementary Movie 5

Description: Raw data kymograph (98.75s long) movie bacteriorhodopsin WT. The dark and green light activation periods are indicated by the alternating black and green bar below the movie. Movie acquisition parameters: 25nm / 150pixels, 600 lines / second.
