# Supplementary material for: Single molecule kinetics of bacteriorhodopsin by HS-AFM
Source: Nat Commun. 2021 Dec 10;12:7225. doi: 10.1038/s41467-021-27580-2 (PMC8664958; doi:10.1038/s41467-021-27580-2)
Supplement: Supplementary file 9 — Reporting Summary [file 41467_2021_27580_MOESM9_ESM.pdf]

nature portfolio

Corresponding author(s): Simon Scheuring
Last updated by author(s): Nov 19, 2021

Reporting Summary

Nature Portfolio wishes to improve the reproducibility of the work that we publish. This form provides structure for consistency and transparency in reporting. For further information on Nature Portfolio policies, see our Editorial Policies and the Editorial Policy Checklist.

Statistics

For all statistical analyses, confirm that the following items are present in the figure legend, table legend, main text, or Methods section.

n/a	Confirmed	
☐	☒	The exact sample size (*n*) for each experimental group/condition, given as a discrete number and unit of measurement
☐	☒	A statement on whether measurements were taken from distinct samples or whether the same sample was measured repeatedly
☒	☐	The statistical test(s) used AND whether they are one- or two-sided<br>*Only common tests should be described solely by name; describe more complex techniques in the Methods section.*
☒	☐	A description of all covariates tested
☒	☐	A description of any assumptions or corrections, such as tests of normality and adjustment for multiple comparisons
☐	☒	A full description of the statistical parameters including central tendency (e.g. means) or other basic estimates (e.g. regression coefficient) AND variation (e.g. standard deviation) or associated estimates of uncertainty (e.g. confidence intervals)
☒	☐	For null hypothesis testing, the test statistic (e.g. *F*, *t*, *r*) with confidence intervals, effect sizes, degrees of freedom and *P* value noted<br>*Give P values as exact values whenever suitable.*
☒	☐	For Bayesian analysis, information on the choice of priors and Markov chain Monte Carlo settings
☒	☐	For hierarchical and complex designs, identification of the appropriate level for tests and full reporting of outcomes
☒	☐	Estimates of effect sizes (e.g. Cohen's *d*, Pearson's *r*), indicating how they were calculated

Our web collection on statistics for biologists contains articles on many of the points above.

Software and code

Policy information about availability of computer code

Data collection	Igor Pro version 6.37 was used for data collection in the High-Speed AFM microscope
Data analysis	Data analysis was done with customs scripts in Matlab R2018b and ImageJ FIJI v1.52. All codes are either published, Ref 36-37, 40 (https://github.com/George-R-Heath/AFM-LineScan-Analyser) and 43 (STaSi algorithm), or available to editors and reviewers

For manuscripts utilizing custom algorithms or software that are central to the research but not yet described in published literature, software must be made available to editors and reviewers. We strongly encourage code deposition in a community repository (e.g. GitHub). See the Nature Portfolio guidelines for submitting code & software for further information.

Data

Policy information about availability of data

All manuscripts must include a data availability statement. This statement should provide the following information, where applicable:

- Accession codes, unique identifiers, or web links for publicly available datasets
- A description of any restrictions on data availability
- For clinical datasets or third party data, please ensure that the statement adheres to our policy

All data supporting the findings of this study are available within the article and its supplementary information files. Additional information and relevant raw data are available from the corresponding author upon reasonable request and at the earliest convenience. Protein data bank codes used are: PDB 6RNJ 10.2210/pdb6RNJ/pdb, PDB 6RPH 10.2210/pdb6RPH/pdb and PDB 6RQP 10.2210/pdb6RQP/pdb.

Field-specific reporting

Please select the one below that is the best fit for your research. If you are not sure, read the appropriate sections before making your selection.

☒ Life sciences ☐ Behavioural & social sciences ☐ Ecological, evolutionary & environmental sciences

For a reference copy of the document with all sections, see nature.com/documents/nr-reporting-summary-flat.pdf

Life sciences study design

All studies must disclose on these points even when the disclosure is negative.

Sample size	No statistical methods were used to determine sample size. Experiments performed under continuous light were analyzed with more than 450 events. Short light pulses experiments were analyzed from bR-WT 110 events and bR-D96N 67 events. The number of events analyzed are more than sufficient for the exponential fittings applied.
Data exclusions	No data were excluded from the analyses
Replication	All attempts at replication were successful. Data for analysis was recorded in different days and a new sample was prepared each day accordingly with the methods section. All results show statistically similar results.
Randomization	The study did not allocate experimental groups. No randomization was required for the reported experiments
Blinding	Not applicable to this study as there was just one experimental group.

Reporting for specific materials, systems and methods

We require information from authors about some types of materials, experimental systems and methods used in many studies. Here, indicate whether each material, system or method listed is relevant to your study. If you are not sure if a list item applies to your research, read the appropriate section before selecting a response.

Materials & experimental systems

n/a	Involved in the study	
☒	☐	Antibodies
☒	☐	Eukaryotic cell lines
☒	☐	Palaeontology and archaeology
☒	☐	Animals and other organisms
☒	☐	Human research participants
☒	☐	Clinical data
☒	☐	Dual use research of concern

Methods

n/a	Involved in the study	
☒	☐	ChIP-seq
☒	☐	Flow cytometry
☒	☐	MRI-based neuroimaging
